# Understanding Social Determinants of First Nations Health Using a Four-Domain Model of Health and Wellness Based on the Medicine Wheel: Findings from a Community Survey in One First Nation

**DOI:** 10.3390/ijerph19052836

**Published:** 2022-02-28

**Authors:** Bryan Tanner, Sara Plain, Tracey George, Julie George, Christopher J. Mushquash, Sharon Bernards, Melody Morton Ninomiya, Samantha Wells

**Affiliations:** 1 Institute for Mental Health Policy Research, Centre for Addiction and Mental Health, 100 Collip Circle, London, ON N6G 4X8, Canada; sharon.bernards@camh.ca (S.B.); mmortonninomiya@wlu.ca (M.M.N.); samantha.wells@camh.ca (S.W.); 2 E’Mino Bmaad-Zijig Health Centre, Aamjiwnaang First Nation, 1300 Tashmoo Ave, Sarnia, ON N7T 7H5, Canada; splain@aamjiwnaang.ca (S.P.); tgeorge@aamjiwnaang.ca (T.G.); 3 Mental Health, Addiction and Violence Support Program, Kettle & Stony Point Health Services, A-6275 Indian Lane, Kettle & Stony Point First Nation, Lambton Shores, ON N0N 1J1, Canada; julieg@ksphs.on.ca; 4 Department of Psychology, Lakehead University, 955 Oliver Rd, Thunder Bay, ON P7B 5E1, Canada; cjmushqu@lakeheadu.ca; 5 Northern Ontario School of Medicine, Lakehead University, 955 Oliver Rd, Thunder Bay, ON P7B 5E1, Canada; 6 Dilico Anishinabek Family Care, 200 Anemki Pl, Fort William First Nation, ON P7J 1L6, Canada; 7 Centre for Rural and Northern Health Research, Thunder Bay, ON P7B 5E1, Canada; 8 Thunder Bay Regional Health Sciences Centre, 980 Oliver Rd, Thunder Bay, ON P7B 6V4, Canada; 9 Thunder Bay Regional Health Research Institute, 1040 Oliver Rd, Thunder Bay, ON P7B 7A5, Canada; 10 Health Sciences, Wilfrid Laurier University, 75 University Ave W, Waterloo, ON N2L 3C5, Canada; 11 Campbell Family Mental Health Research Institute, Centre for Addiction and Mental Health, 33 Russel Street, Toronto, ON M5S 3M1, Canada; 12 Dalla Lana School of Public Health, University of Toronto, 150 College St, Toronto, ON M5T 3M7, Canada; 13 Department of Epidemiology and Biostatistics, Western University, 1151 Richmond St., London, ON N6A 3K7, Canada; 14 Department of Psychiatry, University of Toronto, 27 King’s College Circle, Toronto, ON M5S 1A1, Canada; 15 School of Psychology, Deakin University, 664 Collins Street, Melbourne, VIC 3217, Australia

**Keywords:** First Nations, colonialism, resilience, epidemiology, discrimination, Medicine Wheel

## Abstract

We examined the explanatory roles of social determinants of health (SDOH) for First Nations people using a four-domain model of health and wellness based on the Medicine Wheel (i.e., physical, mental, emotional, and spiritual health), including colonial-linked stressors (i.e., historical trauma, childhood adversities, racial discrimination) and cultural resilience factors (i.e., cultural strengths, traditional healing practices, social support). Data were collected in partnership with a First Nation in Ontario, Canada in 2013 through a community survey (*n* = 194). For each outcome (physical, mental, emotional, and spiritual health), a modified Poisson regression model estimated prevalence ratios for the SDOH, adjusting for age, sex, education, and marital status. Negative associations were found for historical trauma with physical, mental, emotional, and spiritual health; for childhood adversities with mental health; and for racial discrimination with physical, mental, and emotional health. Positive associations were found for cultural strengths with physical, mental, and emotional health and for social support with physical, mental, emotional, and spiritual health. We observed negative associations between use of traditional healing practices and mental and emotional health. Our findings suggest that these SDOH may play important roles in relation to wellness through associations with the domains of health modelled by the Medicine Wheel.

## 1. Introduction

In the last twenty years, researchers have increasingly examined the roles that social determinants of health (SDOH) play in shaping health outcomes for First Nations peoples [1,2,3,4]. In particular, systematic discrimination arising from colonization by European settlers of the land that is now known as Canada has been shown to have substantial negative intergenerational health effects, while First Nations culture and cultural identity have emerged as crucial factors in promoting wellness [1,5,6,7,8,9].

In Canada, First Nations peoples have faced systematic attempts at coerced assimilation into White Settler society through acts and policies designed to curtail their autonomy and cultural identity [2,3,4,8]. Justified by discriminatory laws, such as the 1876 Indian Act, First Nations communities have been exposed to several discriminatory and traumatic events and policies at the behest of the Canadian government, including forced resettlement, land seizure, suppression of language and cultural practices, residential and day schooling, racialized policing, and racist family separation policies, such as the 60s scoop. In many communities, these traumatic events have caused immense disruption in First Nations people’s sense of identity, knowledge and understanding of roles and responsibilities, family ties, and social and community connectedness, in turn contributing to the large health disparities that exist today between First Nations and Settler populations [2,3,4]. Studies on the health impacts of these adversities have focused on how events, such as residential schooling, the 60s scoop, and violence, give rise to an intergenerational form of post-traumatic stress known as historical trauma [5,6,10,11,12]. In addition to shaping health outcomes directly, historical trauma has been linked to increased exposure to additional life stressors, including adverse experiences during childhood as well as increased exposure to racial discrimination as an adult [13].

Despite these adversities, many of the first peoples of Canada have continued to thrive, demonstrating great resilience in the face of the attempted destruction of their ways of life [7,14]. For First Nations peoples, ensuring resilience in light of these adversities can involve drawing upon First Nations identity, which is inextricably linked to history and culture [7]. Several factors associated with First Nations cultural resilience have been identified as key determinants of wellness, including access to and participation in cultural practices [15,16], use of traditional healing systems [7], and availability of strong social bonds [17,18].

Research on these SDOH has largely focused on understanding associations with a narrow set of poor health outcomes, including depression, substance use, and suicidal behaviours [1,5,19]. Conversely, little attention has been given to exploring the impact of these SDOH from a distinctly First Nation’s perspective. One important First Nation’s framework that remains underutilized in the epidemiological literature is the Medicine Wheel. The Medicine Wheel represents First Nations teachings on interconnectedness and worldviews, including aspects of health and wellness. Though these teachings vary greatly, in many versions of the Medicine Wheel, health is conceptualized in terms of a state of wellness that is attained through balance of physical, mental, emotional, and spiritual domains. Each of these domains has distinct implications for the wellbeing of an individual. According to Dapice [20], physical health refers to the functioning of the physical body and associated health conditions (i.e., cardiovascular disease; obesity). Mental health comprises aspects related to cognitive functioning, which is strengthened by traditional teachings and experiential learning, communication, and use of First Nations languages. Emotional health comprises affective or mood elements. Lastly, spiritual health relates to connections to the Creator and creation, the absence of which is associated with feelings of hopelessness and despair. Furthermore, the Medicine Wheel emphasizes connections beyond the individual as key components to wellness, including connections within one’s family, community, and environment [21].

Given the importance of the Medicine Wheel as a learning paradigm to many First Nations peoples, the lack of epidemiological research drawing on this framework is a notable oversight. Thus, in this study, we used a four-domain model of health and wellness based on the Medicine Wheel to re-examine several known SDOH. These SDOH include stressors stemming from colonialism (i.e., historical trauma, childhood adversities, and racial discrimination) and cultural resilience factors (i.e., cultural strengths, use of traditional healing practices, and social support).

## 2. Materials and Methods

### 2.1. Project Overview and Data Source

This study was part of a secondary analysis of cross-sectional survey data collected with Aamjiwnaang First Nation, an Anishinaabe community centered on a peri-urban reserve near Sarnia, Ontario, Canada. Aamjiwnaang First Nation includes approximately 2400 members, including 850 who live on the reserve. Like many First Nations, Aamjiwnaang has dealt with discriminatory policies and actions stemming from colonization. Community specific risk factors also exist; for instance, environmental degradation due to its location within Canada’s Chemical Valley has been cited as a key determinant of health for community members. Nevertheless, Aamjiwnaang remains a vibrant and robust community. Control of essential public services is exercised by the band, and strong connections are maintained with nearby communities, including other First Nations, as well as with the city of Sarnia [22].

Through a unique collaboration between community-based healthcare workers and researchers at the Centre for Addiction and Mental Health (CAMH), a community survey was conducted in 2013 as part of Researching Health in Ontario Communities (RHOC). RHOC was a multisite, collaborative, community-based research project led by researchers at CAMH to gain an understanding of mental health, substance use, and violence challenges, their co-occurrence, and the capacity of communities to respond to these challenges in eight underserved communities in southern Ontario, Canada, including two First Nations. The surveys conducted in the participating First Nations were adapted to include questions that were important to the communities, including measures of historical loss and racism, community-specific strengths and challenges, and use of traditional healing methods.

The Aamjiwnaang First Nation health committee provided advice and guidance regarding all aspects (including data collection approaches and measures) and through all stages of the project. Authors of this article include Aamjiwnaang First Nation healthcare leaders (S.P., T.G.), as well as Indigenous (J.G., C.M.) and non-Indigenous (B.T., M.M.N., S.B., S.W.) researchers from outside the community.

### 2.2. Recruitment and Data Collection Procedures 

Recruitment of participants involved both a simple random sample and a convenience sample. For the simple random sample, invitations were sent by mail to 376 band members aged 18 years and over, selected at random from band membership lists. Follow-up telephone calls and a visit by community research assistants were done to encourage participation. Of the 376 community members contacted, 104 completed some or all of the questionnaire, resulting in a response rate of approximately 28%. At the request of the Aamjiwnaang Health Committee, survey participation was open to all adult band members. As such, posters were displayed around the community inviting anyone interested to participate. The convenience sample included an additional 137 participants. The total sample (*n* = 241) comprised approximately 10% of the total band membership of ~2400 individuals. Data were collected via an English-language questionnaire offered on laptop or paper; this questionnaire took approximately one hour to complete. Data collection was facilitated by use of the CAMH Mobile Lab, a mobile research facility equipped with laptops for data collection. Research staff included local community members trained in research and data collection procedures. Participants were remunerated with a gift certificate valued at CAD 25.

### 2.3. Measures

Four Domains of Health: Participants were asked to separately rate their physical, mental, emotional, and spiritual health, each rated using a single Likert-style item. Response options for each item included poor, fair, good, very good, and excellent. Responses were dichotomized in the usual fashion for measures of self-rated health (poor/fair vs. good/very–good/excellent) [23]. The analyses assessed associations of the proposed SDOH with good or better (i.e., good/very good/excellent) health status, with each of the four domains of health treated as separate outcomes. As such, other aspects of the Medicine Wheel, including the central role of balance between the four domains, were not addressed.

Socio-demographics: Variables included measures of sex (male/female), age (in years), education (less than high school/high school or greater), and marital status (neither married nor common law/married or common law). These socio-demographic variables were included as covariates due to their conceptual importance as determinants of health status for First Nations peoples [4,24].

Stressors: Historical trauma was assessed using Whitbeck and colleagues’ [25] Historical Loss scale, a twelve-item instrument designed to measure occurrence of thoughts of historical losses (e.g., of land; of culture) due to social and cultural disruption arising from colonialization (α = 0.93). Response options ranged from “never” to “daily”. Childhood adversities were measured using the eight-item childhood trauma measure from Turner, Wheaton, and Lloyd’s [26] lifetime trauma scale and modelled as a count variable (α = 0.71). The measure was adapted by the survey team to make it more locally relevant; changes included removing one item (“Did you have to do a year of school over again”) and changing the wording on another (“Were you sent away from home because you did something wrong?” to “Were you sent away from home?”). Further, two items (“Were you ever physically abused by someone close to you?” and “Were you ever physically abused by someone not close to you”) replaced “Were you regularly physically abused by one of your parents?”. Racial discrimination was assessed using the ten-item Measure of Indigenous Racism Experience scale [27] (α = 0.89).

Cultural Resilience Factors: Cultural strengths were assessed using a four-item scale adapted from a thirteen-item checklist featured in the 2008/2010 iteration of the First Nations Regional Health Survey [28]. A measure was created for the present analyses based on whether participants agreed that the following four strengths were a characteristic of their community: “use of First Nations language”, “the natural environment”, “traditional ceremonial activities and awareness of First Nations culture”, and “Elders”. Affirmative responses were summed to create a count variable that ranged from 0 to 4 as done previously by Spence and colleagues [29]. Use of traditional healing practices was assessed using a pair of measures capturing use of traditional healers and use of traditional medicines. As substantial overlap was identified in the use of the healers and medicines within the sample, we chose to combine the two measures into a single composite measure. Thus, participants who answered yes to either were coded as “uses traditional healing”; else, participants were coded as “does not use traditional healing”. Social support was measured using a five-item instrument developed by Schieman [30] to measure perceived social support (α = 0.86). While the original instrument measured social support received from friends and family, the survey team adapted the wording to reflect social support received from family, friends, neighbours, and their community. 

### 2.4. Analytic Strategy 

All analyses were done using Stata 14 software. Descriptive statistics were calculated for all variables. These included means and standard deviations for continuous or count variables and proportions for discrete variables. Modified Poisson regression was used to estimate unadjusted (PR) and adjusted prevalence ratios (aPR) assessing the relationships between the independent variables and each domain of health [31]. Similar to odds ratios, PRs equaling one suggest a null association, while values lower than one and greater than one represent negative and positive associations, respectively.

To calculate adjusted prevalence ratios (aPR), separate multivariable models were fit for each of the stressors and cultural resilience factors, adjusting for socio-demographics (i.e., sex, age, education, and marital status). To improve interpretability, the multi-item measures of historical trauma, racial discrimination, and social support were rescaled with means = 0 and standard deviations = 1 for the regression analyses; estimates for these variables are interpretable as the estimated percentage change in prevalence per change in standard deviation of the independent variable. Scores for missing items on these multi-item measures were prorated, with the mean score calculated from all non-missing items if 20% or fewer items were missing. Participants with missing responses for more than 20% of items on the multi-item measures or missing responses on other questions were excluded from the analyses, resulting in an analyzed sample of 194 participants.

## 3. Results

Full characteristics of the sample are described in Table 1. A majority of participants rated their health as good or better across each of the four domains (63% for physical health, 73% for mental health, 68% for emotional health, and 71% for spiritual health). Women made up a majority of the sample (61%). Average age was slightly less than 39 years old (mean = 38.9; standard deviation = 14.7). The sample consisted of several generations of community members (range: 18 to 77), including representation from younger adults (38% age 18 to 30), adults (54% age 31 to 59), and Elders (9% age 60+). A large majority of participants had at least a high school education (74%). Slightly less than half (45%) of participants were married or living with a common law partner.

As shown in Table 2, historical trauma showed consistent negative associations with each of the four domains of health, including physical (aPR: 0.87, 95% CI: 0.78 to 0.98), mental (aPR 0.91, 95% CI: 0.83 to 0.98), emotional (aPR: 0.83, 95% CI: 0.75 to 0.91), and spiritual health (aPR: 0.90, 95% CI: 0.82 to 0.99). Exposure to racial discrimination was associated with decreased likelihood of reporting good or better physical (aPR: 0.87, 95% CI: 0.78 to 0.97), mental (aPR: 0.86, 95% CI: 0.79 to 0.94), and emotional health (aPR: 0.83, 95% CI: 0.75 to 0.91). Childhood adversities were associated with decreased likelihood of reporting good or better mental health (aPR: 0.95, 95% CI: 0.91 to 0.99).

Among the cultural resilience factors, each cultural strength identified within the community was associated with a higher likelihood of reporting good or better physical (aPR: 1.08, 95% CI: 1.01 to 1.16), mental (aPR: 1.06, 95% CI: 1.00 to 1.13), and emotional health (aPR: 1.09, 95% CI: 1.02 to 1.16). Use of traditional healing was associated with lower likelihood of reporting good or better mental (aPR: 0.79, 95%: 0.65 to 0.96) and emotional health (aPR: 0.74, 95% CI: 0.6 to 0.91). Finally, social support was associated with higher likelihood of reporting good or better physical (aPR: 1.17, 95% CI: 1.02 to 1.33), mental (aPR: 1.25, 95% CI: 1.10 to 1.41), emotional (aPR: 1.36, 95% CI: 1.17 to 1.58), and spiritual health (aPR: 1.18, 95% CI: 1.05 to 1.35).

## 4. Discussion

In this study, we considered the health impacts of stressors stemming from colonialism and culture resilience factors in light of a wellness-oriented four-domain model of health based on the Medicine Wheel. Our findings suggest that stressors stemming from colonialism and cultural resilience factors may be important in shaping wellness by broadly influencing the multiple domains of health included in the Medicine Wheel.

Taken together, our findings for the stressors reflect previous research that has identified these explanatory factors as important determinants of health outcomes, such as depressive symptomology, substance use, and suicidal behaviours [1,6,32], but through a wellness-oriented lens incorporating the four dimensions of health based on the Medicine Wheel. Like many First Nations people, members of Aamjiwnaang First Nation have dealt with sustained assaults on their cultural identity through discriminatory acts and policies, such as residential and day schools, land seizures, and suppression of cultural practices. In the present study, we found that the historical trauma stemming from these events was associated with a decreased likelihood of reporting good or better health across each of the four health domains. This finding suggests that these historic stressors may have lasting intergenerational implications for wellness by way of broad associations with contemporary physical, mental, emotional, and spiritual health. Similar associations were found for racial discrimination although an observed negative association with spiritual health was not statistically significant. This finding reflects favorably on past research that has found racial discrimination to be broadly associated with poorer physical and psychological health [33,34,35]. For the measure of childhood adversities, a statistically significant association was found with mental health status. This finding is consistent with previous research showing a strong link between childhood adversities and psychological health [36]. One potential explanation for this finding is that these adversities act as a disrupting influence during the key phase of life when cultural traits that promote positive mental health (e.g., use of language; traditional teachings) are transmitted to a new generation [37].

Turning to resilience measures, our findings suggest important positive roles for social and cultural bonds in relation to health and wellness. Greater perception of cultural strengths within the community was significantly associated with good or better physical, mental, and emotional health. Furthermore, we found that social support was positively associated with each of the four domains of health. Several factors may explain these observed beneficial associations. A concurrent program of qualitative research involving members of Aamjiwnaang First Nation revealed that strong social and cultural ties were articulated as being important for those facing mental health and substance-use challenges [38]. In that work, sources of support with strong social and cultural ties, such as frontline support workers in the community as well as close family and friends, were preferred for their respectful, understanding nature and for helping individuals feel grounded in a strong Anishinaabe cultural identity. Elsewhere, social support has been hypothesized to buffer the negative effects of stressors on health through several distinct pathways, including through promotion of normative health behaviours and reductions in the intensity of negative emotional states [39]. Likewise, availability of strong cultural institutions is believed to offer locations where positive First Nations group identities can be constructed, helping individuals buffer the impacts of stressors on their health [7]. In the present study, it is plausible that these positive attributes may be driving the broadly beneficial associations that were observed for these two cultural resilience factors.

Interestingly, the use of traditional healing practices was observed to be associated with poorer mental and emotional health. This finding runs contrary to culture as wellness discourses, which centre (re)connecting with First Nations cultural practices as foundational elements to ensuring wellness, particularly in light of trauma associated with colonialism [40]. A possible explanation, which was offered by community health workers when interpreting these findings, is that individuals who face mental and/or emotional health problems may be especially likely to seek support in the community and thus be referred to traditional healing programs. As such, the observed relationships may indicate that the goal of ensuring traditional healing supports are accessed by people who need them, especially those facing mental and emotional health challenges, is being met by the community. However, healthcare workers in the community have noted that those experiencing the most significant mental and emotional health challenges are often unengaged with care options within the community. As these individuals may be missed in surveys [41], it is important for readers to consider that the present findings may not fully reflect help seeking among those living with the most significant challenges. As such, further research is needed to better identify experiences seeking care for these individuals. Moreover, the present measures of help seeking may not fully capture the complexity in patterns of social and cultural connections necessary to sustaining wellness.

### Limitations

It is important to recognize that the Medicine Wheel is foremost a paradigm that helps Indigenous learners situate or make sense of the varied and often interconnected processes experienced in their lives, including those related to their health and wellness. Interpretation of findings for each domain must take into account that our outcome model is a rudimentary abstraction of these deep and interconnected processes that cannot be fully captured by four distinct, binary measures. Similar critiques can be made about the conceptualization and measurement of culture, which is a fluid, intangible, and often deeply personal concept that is not easily broken down into distinct, measurable units. As with all quantitative research examining social phenomena, interpretation of quantitative associations is best contextualized by the voices of those represented by the data. For readers interested in further placing our findings in the context of the lived experiences of community members, we suggest qualitative works by Morton Ninomiya [38] and George [42].

As the data were cross-sectional, inferences regarding causality cannot be made from our findings. The inclusion of participants recruited by non-probabilistic convenience sampling and the low response rate among those sampled randomly may be potential sources of selection bias. As Smylie and Firestone [41] noted, non-response in surveys with Indigenous populations may be associated with several known determinants of poorer health, such as poverty, poor literacy, and housing instability, which can lead to underestimation of social and health disadvantage. Furthermore, definitions of each domain of health were not provided to participants, and therefore, meanings of each domain may not be consistent for all participants. Finally, although a relatively large portion of the community participated in the study, the sample size was not sufficient to examine associations within strata of theoretically important third variables (e.g., gender differences; age differences) and may have contributed to low statistical power to detect significant associations. Thus, these findings are likely conservative and should be considered preliminary. Nevertheless, this unique study drawing on a four-domain model based on the Medicine Wheel brings to light important SDOH linked to health and wellness in a novel way.

## 5. Conclusions

While the Medicine Wheel has been previously adapted to measure wellness-oriented outcomes in clinical settings [43], to our knowledge, this was the first study to apply this framework to understanding health in a community sample. Our findings suggest that these SDOH may be important in shaping health outcomes for First Nations peoples by influencing each of the health outcome components, namely physical, mental, emotional, and spiritual health. While our outcome measures offer a starting point for examining health as understood in the Medicine Wheel, more work is needed to better integrate aspects of this framework that were left unaddressed in our analysis. Specifically, future research should explore ways to integrate the concept of balance into measurement of health across the four domains as well as the interpersonal aspects of wellness, such as familial, community, and environmental connections.

## Figures and Tables

**Table 1:** Descriptive characteristics for the total sample and by outcome.

Variable	Sample (*n* = 194)	Physical Health		Mental Health		Emotional Health		Spiritual Health	
		Good or Better (*n* = 123)	Poor or Fair (*n* = 71)	Good or Better (*n* = 141)	Poor or Fair (*n* = 53)	Good or Better (*n* = 132)	Poor or Fair (*n* = 62)	Good or Better (*n* = 137)	Poor or Fair (*n* = 57)
Socio-Demographics									
Sex									
Female, *n (%)*	119 (61%)	72 (61%)	47 (39%)	82 (69%)	37 (31%)	72 (61%)	47 (39%)	79 (66%)	40 (34%)
Male, *n (%)*	75 (39%)	51 (68%)	24 (32%)	59 (79%)	16 (21%)	60 (80%)	15 (20%)	58 (77%)	17 (23%)
Age, *mean (SD)*	38.9 (14.7)	37.2 (14.4)	41.9 (14.8)	40.2 (14.6)	35.3 (14.5)	40.6 (14.2)	35.3 (15.1)	40.2 (14.6)	35.6 (14.4)
Education									
High School or Greater, *n (%)*	144 (74%)	92 (64%)	52 (36%)	112 (78%)	32 (22%)	106 (74%)	38 (26%)	107 (74%)	37 (26%)
Less than High School, *n (%)*	50 (26%)	31 (62%)	19 (38%)	29 (58%)	21 (42%)	26 (52%)	24 (48%)	30 (60%)	20 (40%)
Marital Status									
Married/Common Law, *n (%)*	86 (45%)	52 (60%)	34 (40%)	68 (79%)	18 (21%)	67 (78%)	19 (22%)	66 (77%)	20 (23%)
Not Married/Common Law, *n (%)*	108 (55%)	71 (66%)	37 (34%)	73 (68%)	35 (32%)	65 (60%)	43 (40%)	71 (66%)	37 (34%)
Stressors									
Historical Trauma, *mean (SD)*	34.7 (14.2)	32.8 (13.3)	38 (15.1)	33.5 (14.2)	37.8 (13.8)	32.40 (14.3)	39.6 (12.7)	33.4 (14)	37.6 (14.3)
Childhood Adversities, *mean (SD)*	3.1 (2.2)	3 (2.1)	3.2 (2.3)	2.9 (2.1)	3.8 (2.3)	2.9 (2.1)	3.7 (2.2)	3.1 (2.2)	3.3 (2.2)
Racial Discrimination, *mean (SD)*	23.4 (8.3)	22.4 (8.3)	25.3 (8.2)	22.2 (8)	26.7 (8.3)	22 (7.9)	26.5 (8.5)	22.7 (7.9)	25.2 (9.2)
Resilience Factors									
Cultural Strengths, *mean (SD)*	1.4 (1.3)	1.5 (1.4)	1.2 (1.2)	1.5 (1.4)	1.1 (1.2)	1.6 (1.4)	1.1 (1.2)	1.5 (1.4)	1.2 (1.1)
Uses Traditional Healing									
Yes, *n (%)*	74 (38%)	45 (61%)	29 (39%)	48 (65%)	26 (35%)	44 (59%)	30 (41%)	54 (73%)	20 (27%)
No, *n (%)*	120 (62%)	78 (65%)	42 (35%)	93 (78%)	27 (22%)	88 (73%)	32 (27%)	83 (69%)	37 (31%)
Social Support, *mean (SD)*	20.2 (4.1)	20.8 (3.9)	19.2 (4.4)	21 (3.6)	18 (4.7)	21.1 (3.6)	18 (4.4)	20.1 (3.9)	18.7 (4.5)

SD: standard deviation.

**Table 2:** Associations of the stressors and cultural resilience factors with the four domains of health (*n* = 194).

	Unadjusted Associations		Adjusted Associations	
	PR (95% CI)	*p*-Value	aPR (95% CI)	*p*-Value
Physical Health				
Historical Trauma	0.87 (0.78 to 0.98)	0.02	0.87 (0.78 to 0.98)	0.02
Childhood Adversities	0.98 (0.94 to 1.04)	0.55	0.98 (0.93 to 1.04)	0.52
Racial Discrimination	0.88 (0.79 to 0.98)	0.02	0.87 (0.78 to 0.97)	0.02
Cultural Strengths	1.07 (0.99 to 1.16)	0.06	1.08 (1.01 to 1.16)	0.03
Uses Traditional Healing	0.94 (0.75 to 1.17)	0.56	0.90 (0.72 to 1.13)	0.38
Social Support	1.16 (1.02 to 1.32)	0.02	1.17 (1.02 to 1.33)	0.03
Mental Health				
Historical Trauma	0.92 (0.84 to 1.00)	0.06	0.91 (0.83 to 0.98)	0.02
Childhood Adversities	0.95 (0.91 to 0.99)	0.02	0.95 (0.91 to 0.99)	0.03
Racial Discrimination	0.86 (0.78 to 0.94)	0.001	0.86 (0.79 to 0.94)	0.001
Cultural Strengths	1.07 (1.01 to 1.13)	0.03	1.06 (1.00 to 1.13)	0.04
Uses Traditional Healing	0.84 (0.69 to 1.02)	0.07	0.79 (0.65 to 0.96)	0.02
Social Support	1.24 (1.10 to 1.39)	<0.001	1.25 (1.10 to 1.41)	<0.001
Emotional Health				
Historical Trauma	0.85 (0.77 to 0.94)	0.001	0.83 (0.75 to 0.91)	<0.001
Childhood Adversities	0.95 (0.90 to 0.99)	0.02	0.96 (0.91 to 1.00)	0.05
Racial Discrimination	0.83 (0.75 to 0.93)	0.001	0.83 (0.75 to 0.91)	<0.001
Cultural Strengths	1.09 (1.02 to 1.17)	0.01	1.09 (1.02 to 1.16)	0.008
Uses Traditional Healing	0.81 (0.65 to 1.01)	0.06	0.74 (0.60 to 0.91)	0.004
Social Support	1.33 (1.16 to 1.52)	<0.001	1.36 (1.17 to 1.58)	<0.001
Spiritual Health				
Historical Trauma	0.92 (0.83 to 1.01)	0.07	0.90 (0.82 to 0.99)	0.03
Childhood Adversities	0.98 (0.94 to 1.03)	0.45	0.99 (0.95 to 1.03)	0.67
Racial Discrimination	0.91 (0.83 to 1.01)	0.07	0.91 (0.83 to 1.00)	0.05
Cultural Strengths	1.06 (0.99 to 1.13)	0.05	1.06 (1.00 to 1.12)	0.05
Uses Traditional Healing	1.06 (0.88 to 1.27)	0.57	1.01 (0.84 to 1.21)	0.92
Social Support	1.18 (1.04 to 1.32)	0.005	1.18 (1.05 to 1.35)	0.007

PR, prevalence ratio; CI, confidence interval; aPR, adjusted prevalence ratio. Each aPR adjusted for sex, age, education, and marital status only; not adjusted for other variables in the table.

## Data Availability

To ensure participant confidentiality, data are not publicly available.
